# Prevalence and Risk Factors of Ovine and Caprine Fasciolosis in the Last 20 Years in China: A Systematic Review and Meta-Analysis

**DOI:** 10.3390/ani13101687

**Published:** 2023-05-18

**Authors:** Zhuo Lan, Jian Yu, Xinhui Zhang, Aihui Zhang, Ruipeng Deng, Ben Li, Qingbo Lv, Xiaoxiao Ma, Junfeng Gao, Chunren Wang

**Affiliations:** 1 Key Laboratory of Bovine Disease Control in Northeast China, College of Animal Science and Veterinary Medicine, Heilongjiang Bayi Agricultural University, Ministry of Agriculture and Rural Affair, Daqing 163319, China; 2 Nehe Animal Husbandry Technology Promotion Center, Nehe 161300, China; 3 Key Laboratory of Zoonosis Research, Institute of Zoonosis, College of Veterinary Medicine, Jilin University, Ministry of Education, Changchun 130062, China

**Keywords:** fasciolosis, sheep, goat, prevalence, risk factors, meta-analysis

## Abstract

**Simple Summary:**

*Fasciolia hepatica* and *Fasciola gigantica* are widespread and found in the liver and bile ducts of ruminant animals and humans, which poses a major problem in animal husbandry. There are many studies on the prevalence of ovine and caprine fasciolosis in China, but the overall prevalence of fasciolosis and the risk factors are still unclear. This systematic review and meta-analysis was designed to investigate the prevalence and risk factors of ovine and caprine fasciolosis in China. The pooled prevalence of ovine and caprine fasciolosis was 26.00%. Out of all the subgroups, the type of season and sampling years showed the most significant (*p* < 0.05) differences. The results indicated that ovine and caprine fasciolosis was widely distributed, especially in Northwestern China. The risk factors related to the disease are described in this study. Therefore, strategies for ovine and caprine fasciolosis control can be developed based on the epidemic risk factors that are identified in this study, which will thus reduce the prevalence of fasciolosis in China.

**Abstract:**

Fasciolosis is a significant zoonotic and common parasitic disease for animals and humans, creating public health concerns worldwide. This study retrieved articles related to the occurrence of *Fasciola hepatica* and *Fasciola gigantica* in sheep and goats in China by searching five databases: PubMed, ScienceDirect, the Chinese National Knowledge Infrastructure (CNKI), Wanfang Data, and the VIP Chinese Journal Database. A total of 60 valid articles were captured. The pooled prevalence of ovine and caprine fasciolosis was 26.00%. It was also found to be higher in the subgroups of Northwest China and Shaanxi Province, as well as in areas with a high altitude, rainfall of ≥800 mm, and temperature ranging between 10 °C and 20 °C. Analysis of the type of season and sampling years showed significant (*p* < 0.05) difference. In other subgroups, sheep (34.74%), hosts aged over 2 years (32.26%), females (48.33%) and free-range animals (26.83%) showed a higher disease prevalence. These results indicated that ovine and caprine fasciolosis was widely distributed, especially in Northwest China. The sampling years and the type of season are risk factors for the prevalence of ovine and caprine fasciolosis. Therefore, strategies for ovine and caprine fasciolosis control should be developed based on these epidemic risk factors, which will reduce the prevalence of fasciolosis in China.

## 1. Introduction

*Fasciola hepatica* and *Fasciola gigantica* parasitize the liver and bile ducts of herbivorous mammals and humans; however, they most commonly occur in sheep, goats, and cattle [1]. *F. hepatica* occurs mainly in Europe, the Americas and Oceania, and *F. gigantica* occurs mainly in Africa and Asia; together, they are well-known pathogens that cause fasciolosis [2]. In China, *F. hepatica* is distributed mainly in temperate areas while *F. gigantica* occurs primarily in tropical zones. In China, *F. hepatica* has been found in almost all provinces, while *F. gigantica* has only been found in southern area [3]. This disease is primarily known for its high burden and associated economic impact on livestock. The infection of fasciolosis manifests in three phases: acute, sub-clinical, and chronic. Often, the symptoms of acute infection in animals associated with the migration of immature fluke are abdominal pain, lethargy, reduced appetite for grazing, and even death (for heavily infected animals). Sub-clinical disease is slightly more delayed than acute disease, and it presents itself as hemorrhagic anemia. Chronic fasciolosis is caused by the blood-feeding activity of adults, and it presents through symptoms such as low body weight, poor quality fleece, anemia and also severe swelling under the jaw (bottle jaw) [4,5]. This disease can persist for years before being noticed [6]. The detection of *Fasciola* spp. infection is common during the postmortem examination of animals and it is also occasionally found in humans; therefore, it has great impact on animal and human health.

Notably, fasciolosis is the most widely distributed trematode disease reported in over 81 countries around the world [7,8]. For example, a total of 305 sheep flocks were selected in Ireland and the national prevalence of *F. hepatica* was found to be 50.4% [9]. In Mexico, a total of 1070 serum and fecal samples were analyzed for the IgG1 antibodies and coproantiges that are present in goat fasciolosis; following this, a prevalence of 73.46% was found with serological ELISA, and a percentage of 77.20 was found with coproantigen ELISA [10]. In China, 1092 sheep on the Qinghai–Tibet plateau were examined with ELISA kits, and 405 (37.09%) sheep were found to be positive [11]. Wang and Xu reported that 270 goats, which were examined randomly in Qinghai, and 179 goats were suffering from fasciolosis and had a high infection rate (66.30%) [12]. The wide distribution of *Fasciola* is mainly caused by its intermediate host, which is the snail. Furthermore, *Galba pervia*, *Radix cucunorica,* and *Radix swinhoei* are the dominant host snails in China [13,14,15]. Natural conditions and the ecological environment can affect the reproduction of snails, and they can also affect the development of liver fluke in vitro. Certain studies have shown that the climate can affect snail reproduction; furthermore, low-lying and humid swamps, as well as areas with high rainfall and thick soil layers are conducive to snail reproduction, which result in a higher infection rate of *Fasciola*. However, animals in many areas suffer from fasciolosis. According to the statistics, the annual losses caused by fasciolosis are estimated at USD 3.2 billion worldwide [16]. One of the reasons for this is that the quality of biological products such as the meat and milk of these animals will decline because of fasciolosis. Hence, it can be seen that fasciolosis not only seriously threatens the health of animals, but also affects the production performance of ruminants, as well as the development of the animal husbandry economy.

Generally, in all susceptible animals, sheep and goat can provide meat, milk, and wool for human beings, thus they are important livestock in China. With an increase in the human population, the requirements for sheep by products have been elevated worldwide [17]. China is the largest sheep raising country in the world. Thus, investigating the prevalence of ovine fasciolosis in China and identifying the potential risk factors would provide valuable information for researchers and farmers. However, currently, no overall survey has been conducted on ovine fasciolosis in China. In order to better comprehensively understand the occurrence of ovine and caprine fasciolosis and the factors that may affect the prevalence of ovine fasciolosis, a systematic review and meta-analysis covering the last 20 years was performed. 

## 2. Materials and Methods

### 2.1. Search Strategy and Study Selection

This study was performed according to preferred reporting items for systematic review and meta-analysis (PRISMA) guidelines [18]. PubMed (https://pubmed.ncbi.nlm.nih.gov/), ScienceDirect (https://www.sciencedirect.com/), Chinese National Knowledge Infrastructure (CNKI; https://www.cnki.net/), Wanfang Data (http://www.wanfangdata.com.cn/index.html), and the VIP Chinese Journal Database (http://www.cqvip.com/) were used to search for publications regarding *F. hepatica* and *F. gigantica* in sheep and goats. All databases accessed on 25 August 2022. We retrieved all the published papers on the fasciolosis in sheep in China from the date of the earliest literature publication to 25 August 2022. The terms “*Fasciola hepatica*”, “*Fasciola gigantica*”, “fasciolosis”, “Sheep”, “Goat” and “China” were used as the keywords for advanced search, and they were set to be use as synonyms for the purposes of expanding the area of the precise search. All retrieved articles were sent to EndNote X9 and duplicates were removed. The languages were restricted to Chinese and English. Then, we applied the following inclusion criteria: (1) the studies must include prevalence on the presence of fasciolosis in sheep or goat in China; (2) the studies must provide the total number and disease-positive numbers of sheep or goats; (3) the study sample size must be greater than 30; and (4) the study design must be cross-sectional and must provide raw data for epidemiological analysis. Articles were excluded if they were not compatible with these standards. Additionally, we did not contact the original authors to obtain more information, nor did we count unpublished studies.

### 2.2. Data Extraction

We extracted the information from the obtained studies based on the standardized data collection forms in Microsoft EXCEL (v 16.32; Microsoft, Redmond, WA, USA). The information was recorded as follows: first author; publication year; sampling year; the geographical region of the study (including province, administrative regions, and the municipalities directly under the central government); altitude (as classified by Arestegui et al. [19]); rainfall; temperature; season; ovine and caprine species (in both sheep and goats); age; sex; detection method; sampling method; and the total examined number and disease positive number of sheep or goats. The species of *Fasciola* was also counted. If the samples were analyzed via several methods, the data obtained from microscopy were the first priority. This was useful for reducing heterogeneity. 

### 2.3. Quality Assessment

The quality of the selected publications was scored based on the grading of recommendations assessment (GRADE) criteria [20]. The score was then used to rate the quality of the studies. Briefly, a study was awarded one point if a factor from the following list was included: sampling time with specific sampling year; a sampling number greater than 60; sampling method (random or not random); test method; and five more potential risk factors. Additionally, the total score ranged from 0 to 5, and these were classified into three levels. Articles with a total score of 4 or 5 were classified as high-quality, articles with a score of 2 or 3 were classified as medium-quality, and articles with a score of 0 or 1 were classified as low-quality.

### 2.4. Statistical Analysis

All quantitative analyses were performed by the “Meta” package (v 4.11-0) from R Studio software (v 1.2.5019). To find the most suitable model for the data, five methods of fitting were used for the normally distributed data before meta-analysis, including original rate (PRAW), logarithmic conversion (PLN), logit transformation (PLOGIT), arcsine conversion (PAS) and double-arcsine transformation (PFT). If the W-value was close to 1, and the *p*-value was greater than 0.05, then it was close to the Gaussian distribution criterion. Finally, PAS was chosen for the rate conversion data (W = 0.96974 and P = 0.1417). The I^2^ and Cochran’s Q statistical methods were used to quantify the variation [21]. The overall meta-analysis results were presented using forest plots. A pooled estimate of the overall assessment of the meta-analysis and the subgroup analyses were performed by a random-effects model. Additionally, the symmetry of a funnel plot was used to determine the bias for the included studies. Trim and fill analysis, as well as Egger’s test were used to evaluate the publication bias. Sensitivity analysis was used to detect the stability of the results. The heterogeneity was further investigated via subgroup analysis and meta-regression analysis [22,23]. The factors that caused the heterogeneity in this study were examined via an individual model or through multiple-variable models. In the multiple-variable model, the category with the highest prevalence was taken, in the subgroup analysis as the reference group. The factors included geographical region (China is divided into seven administrative regions according to location, including Southwest China, Southeast China, South China, Northeast China, Northwest China, North China, and Central China; a comparison of Northwest China with other regions was also conducted) [24]; altitude (comparison of high altitude that over than 2400 m with low altitude less than 2400 m); sampling season (including spring, summer, autumn, and winter; a comparison of spring with other seasons was also conducted); rainfall (comparison of <800 mm with ≥800 mm); temperature (comparison of <10 °C with 10–20 °C); sampling year (comparison of 2002–2011 with 2012–2022); species (comparison of sheep with goats); age (comparison of >2 years with 0–2 years); sex (comparison of female with male); detection methods (including microscopic examination, autopsy, molecular biology, and immunological test, a comparison of immunological test with other methods was also conducted); feeding management (comparison of free-range animals with stall-feed and half feed); and type of *Fasciola* species (comparison of *F. hepatica* with *F. gigantica*).

## 3. Results

### 3.1. Search Results and Eligible Studies

**Figure 1:**
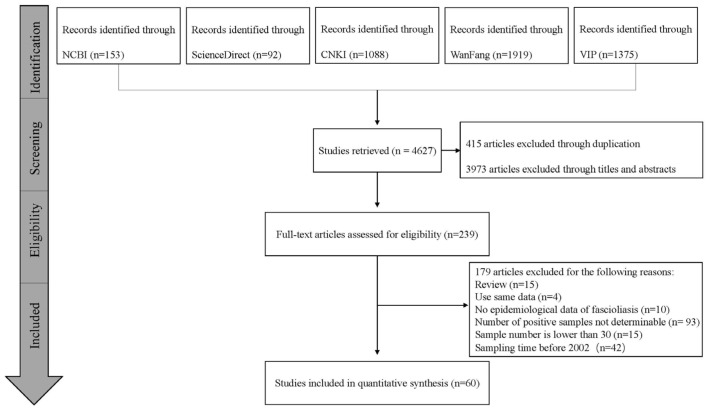
Flow diagram of the literature search and selection of ovine and caprine fasciolosis in the last 20 years in China.

A total of 4627 studies were retrieved from the five databases. According to the inclusion and exclusion criteria, a total of 60 full-text studies comprising 20 high-quality, 39 middle-quality, and 1 low-quality article(s)—which covered 17 provinces—were used for the meta-analysis (Figure 1, Table S1) [11]. 

### 3.2. Publication Bias and Sensitivity Analysis

**Figure 2:**
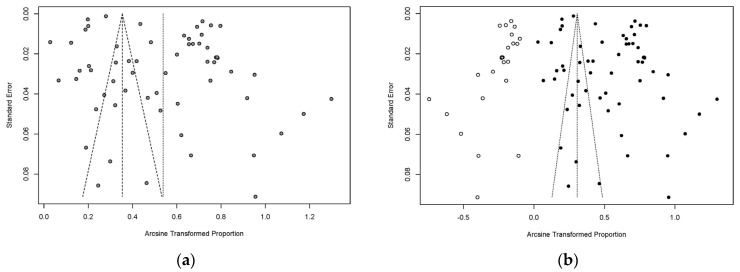
Funnel plot for the examination of publication bias. (**a**) Funnel plot with pseudo 95% confidence interval limits. (**b**) Funnel plot with trim and filling analysis.

**Figure 3:**
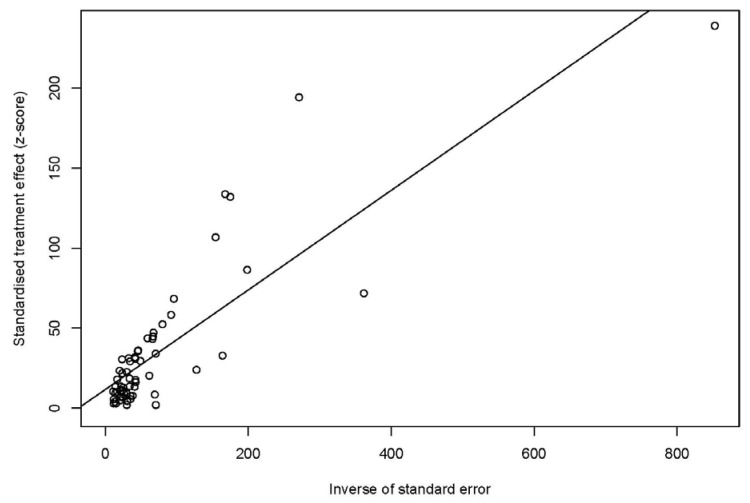
Egger’s test for publication bias.

The funnel plot indicated that a publication bias might exist in the included studies (Figure 2a), and the result of the trim and fill analysis demonstrated that the publication bias only appeared after the addition of the related studies (Figure 2b) [11]. An Egger’s test was conducted to show the extent of the publication bias in this study; the *p*-value was found to be 0.0026 and the value of bias was 11.6737 (Figure 3). The sensitivity test showed that the combined pooled prevalence was not significantly affected by the omission of any of the studies (Figure S1). Thus, the meta-analysis results in this study were reliable. 

### 3.3. Pooling and Heterogeneity Analyses

The forest plot was used to measure and show the heterogeneity index. The random effects model was employed to estimate the prevalence for each subgroup (Figure S2). The pooled prevalence of ovine fasciolosis in the last 20 years in China was 26.00%, and the prevalence of *F. hepatica* and *F. gigantica* were 27.25% (38,912/290,784) and 1.09% (3/267), respectively (Table 1). 

**Figure 4:**
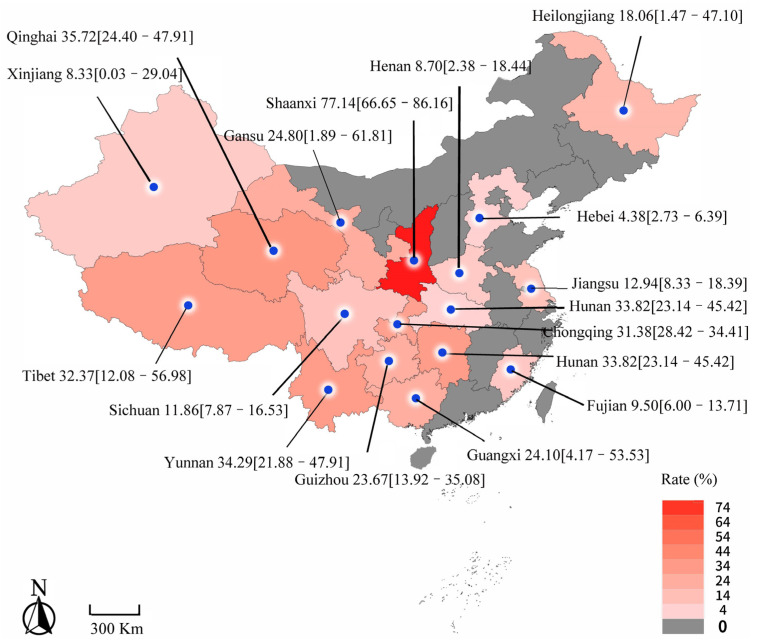
Map of the prevalence of ovine fasciolosis during the last 20 years in China.

The prevalence of ovine and caprine fasciolosis varied between different regions, and it ranged from 4.38% (21/478) to 31.89% (17,531/77,149) (see Table 1). The Shaanxi Province subgroup possessed the highest prevalence (77.14%, 54/70), while Hubei had the lowest prevalence (3.95%, 266/6727) (Figure 4 and Table 1). In the subgroup analysis of altitude, the prevalence of ovine and caprine fasciolosis was 28.23% (5699/42,306) at high altitudes and 23.75% (38/160) at low altitudes. The subgroup analysis based on rainfall showed that ≥800 mm (19.85%, 1791/10,009) demonstrated a higher disease prevalence than <800 mm (15.34%, 24,337/236,786). The temperature of 10–20 °C had a higher disease prevalence (19.85%, 1791/10,009) than 10 °C (13.75%, 5146/41,342). No significant difference existed in subgroups mentioned above. In terms of seasons, the infection rate was highest in spring (36.28%, 5544/75,550), followed by autumn (15.13%, 2550/36,747), summer (11.45%, 552/3183) and winter (5.44%, 6828/74,021). The statistical analysis showed that winter was associated with a lower risk of ovine and caprine fasciolosis (*p* < 0.05). The results mentioned above are shown in Table 1.

As shown in Table 1, the subgroup of sampling years was also analyzed. The percentage of infections in 2002–2011 was 35.75% (9153/29,293), which was different from the years between 2012 and 2022 (20.63%; 28,210/253,080). It can be seen that the difference in sampling years is significant (*p* < 0.05). The analysis for the detection method used showed that the infection rate was highest in the immunological test (43.12%; 4016/8792). The infection rate of microscopy, molecular biology, and slaughter were 30.16% (22,660/234,078), 25.23% (27/107), and 18.11% (14,788/47,546), respectively.

The subgroup analysis based on ovine species showed that the disease prevalence in sheep was higher (34.74%; 14,823/68,473) than in goats (25.41%, 11,861/35,525). As for the prevalence of age, the subgroups of >2 years and 0–2 were 32.26% (8076/18,880) and 22.24% (651/1938), respectively. The analysis of the sex subgroup showed that the disease prevalence in females was 48.33% (8180/18,977) and males 29.54% (248/713). The type of feeding management was also analyzed. The analysis results for free-range animals showed a higher infection rate (26.83%; 19,010/228,303) than in the other two feeding managements. No significant difference was noticed in these groups. These results are shown in Table 1.

## 4. Discussion

Fasciolosis caused by *F. hepatica* and *F. gigantica*, is widely distributed in China [25]. Sheep and goats are important economic animals, thus it necessary to review and assess the prevalence of fasciolosis in these animals in China. To date, this is the first comprehensive systematic review and meta-analysis of the prevalence of ovine fasciolosis and its related risk factors in China. This study may thus provide valuable information for taking measures to prevent fasciolosis. 

Our results showed that there was a diversity in the sampling time subgroup which showed a significant difference (*p* < 0.05). The results showed that the prevalence of ovine and caprine fasciolosis was highest in the years from 2002 to 2011 (35.75%). With the progress of society and the development of the economy, the breeding mode of farms has gradually changed from grazing to semi-house breeding and stall-feed. This change has undoubtedly reduced the prevalence of ovine and caprine fasciolosis, because pasture management factors are a key driver of infection risk [26]. However, fasciolosis still exists, and the prevention and control of the disease are still necessary; thus, more attention should be paid to animal health in China. In the season subgroup, which has a significant difference (*p* < 0.05), the prevalence of fasciolosis was the highest in spring (March–May) (36.28%) and lowest in winter (December-February) (5.44%). In spring, a season that is better for grazing, there was noted to be an increase in the risk of *Fasciola* spp. infection. On the contrary, the temperature needed for fasciolosis is too low in winter, and the grass is withered which is not suitable for grazing. Cruz-Mendoza et al. found that the prevalence of the disease was higher during the warm-wet times than in the cold-dry ones, which was same to the result found in this study [27]. It can be seen that type of season is significantly associated with the prevalence of fasciolosis and is thus identified as a risk factor. 

The prevalence of *F. hepatica* was significantly higher than *F. gigantica* in this study. In addition, 2 articles were related to *F. gigantica,* and 54 studies were on *F. hepatica,* thus showing a huge difference. In accordance with the results of Howell and Williams, *F. hepatica* is the most widespread species, occurring in 70 countries worldwide in mild temperature climates, while *F. gigantica* is present in tropical regions [28]. In China, *F. gigantica* has been reported in Hainan, Yunnan, Anhui and Hebei during the period of 1921 to 2018, while *F. hepatica* has been reported not only in these areas, but also in Jilin, Liaoning, and in many other areas, which have also demonstrated a more common distribution [29].

The result of the region subgroup analysis showed that the highest prevalence of ovine and caprine fasciolosis was 31.89% in Northwest China, and the lowest prevalence was observed in North China (4.38%). However, there was no significant difference in the positive rate of ovine and caprine fasciolosis in the different regions. In addition, in this study, we found a 3.95–77.14% prevalence of ovine and caprine fasciolosis in 17 infected areas. Nonetheless, the current valuable epidemiological data were used to validate the severity of this disease in China, suggesting it having a widespread impact on animal husbandry. Crucially, the prevalence of ovine and caprine fasciolosis in certain areas such as Shaanxi attained high levels. The reason for this maybe in the fact that the Wei River passes the Guanzhong District of Shaanxi, which is rich in water resources with fertile grass that is suitable for snail growth, which would increase the probability of infection. A study showed that relationships can exist between the organization of the hydrological network and the population biology of the fasciolosis disease vector [30]. Thus, appropriate strategies should be custom-designed in areas that have abundant water resources. 

At the altitude level, the results showed that the prevalence of ovine and caprine fasciolosis was much higher in higher altitude without significant difference, which is consistent with Mas-Coma’s study in South America [31]. Therefore, altitude is an important variable that is included in the epidemiological survey of fasciolosis. The effect of the climate environment was analyzed in this study. The results showed that a wet climate (≥800 mm) had a higher disease prevalence than dryer climates. Heavy rainfall creates a more suitable breeding environment for intermediate hosts, thus leading to an increase in *Fasciola* infection [32]. The result of Isah’s study revealed that higher percentages were observed during the early and late rainy season (47.2% and 58.4%) when compared to early and late dry seasons (36.2% and 20.1%) [33], which is consistent with our result. Moreover, the temperature of 10–20 °C (19.85%) also showed a higher disease prevalence in this study. When there is the presence of suitable livestock hosts, an ambient temperature of above 10 °C and sufficient moisture are required for the development and expansion of snail populations [34]. Not only does the aforementioned apply, but warm conditions can also lead to higher infection levels as livestock congregate around the few remaining drinking and grazing areas [28]. Once the drinking water contains infective metacercariae, sheep or goat will be infected. All the reasons mentioned above may increase *Fasciola* spp. infection. Accordingly, reducing grazing and providing animals with safe and clean water are important to help prevent fasciolosis.

The results showed that sheep had higher (34.74%) rates of infection with fasciolosis than goats (25.41%). These results are consistent with the study of Mia et al. [35]. The lifestyles of sheep and goats are different. Goats are picky about grass and eat taller grass. Sheep eat grass on flat slopes and are not picky. Thus, sheep have a greater chance to intake the metacercariae of liver fluke. The present study revealed that animals over 2 years old have a higher infection rate. This is the same in cattle, i.e., the older animals showed a higher prevalence of fasciolosis in Ethiopia [36]. The reason for this may be that older animals take a longer time to graze and thus have more chance of becoming infected. In addition, older animals have lower immunity. The prevalence of females (48.33%) was higher than males (29.54%) This result is similar to the study in Bangladesh. The reason is that high stress during the parturition period may decrease individual immunity, the one with lower immunity may infect disease more susceptibility [35]. Thus, more attention should be paid to monitoring female animals for promoting the prevention and control of fasciolosis.

Although the snail can reflect the environment contamination by *Fasciola* as an epidemiological indicator, certain important factors such as the species, the number and the distribution of the snail affect the prevalence of *Fasciola* [37]. However, there was no analysis on snails in this study. This is because only 8 of the 60 articles in this study mentioned *Fasciola* infection in snails and these studies did not specify the number of infected nor the infection rate. In addition, we searched in five databases the articles related to the prevalence of fasciolosis in snails in China during the last 20 years: only three articles had comprehensive information on the specific infected conditions, which was not enough to conduct any analysis on this as a risk factor. It can be seen that the epidemiological investigation of snail infections should receive more attention in future research.

Four detection methods were mentioned in this study, including immunological test, slaughter, microscopy and molecular biology. The immunological diagnostic method is a kind of method with high sensitivity, especially when investigating early infection. Slaughter is the most accurate clinical diagnostic method, but it is not suitable for large-scale diagnosis and live animal detection. Microscopy and molecular biology assays are also not suitable for early diagnosis. Although the sensitivity and specificity were different in all methods, there was no significant influence in our study. In addition, in the process of fasciolosis diagnosis, appropriate diagnostic methods can be chosen according to different circumstances.

In this study, 20 high-quality articles, 39 middle-quality articles, and 1 low-quality article were analyzed. Moreover, a large sample size was included, which contains the information from 17 provinces from five bibliographic databases. Additionally, we utilized rigorous research methods and conducted in-depth analyses of several subgroups. Therefore, this study reflects the prevalence of ovine and caprine fasciolosis during the last 20 years in China. However, there are certain limitations to this study. First, although we used five databases to retrieve qualified studies, certain studies may still have been missed. Second, only 17 areas are mentioned in this study although there are 31 provinces, cities, and autonomous regions in mainland China; certain areas such as Ningxia, Liaoning, Jiangxi, Jilin, Zhejiang, etc. have epidemic data of fasciolosis before 2002 [38,39,40,41,42], but have no data reported between 2002 and 2022. Bovine fasciolosis was reported in Shanghai and Hainan but there were no reports on ovine and caprine fasciolosis. In addition, although ovine fasciolosis was found in Inner Mongolia, there were no related reports in the five databases. Third, Fujian, Henan, and Chongqing Provinces that were screened in this study only had a single study, which was insufficient. Fourth, certain subgroups such as feeding management, region, and temperature may be identified as risk factors when considering the actual situation, but this was not the case in this study. The reason for this was that there were not enough related data contained in these subgroups, which affected the results to a certain extent.

## 5. Conclusions

In this study, ovine and caprine fasciolosis showed a 26.00% prevalence in the last 20 years in China. Although fasciolosis has decreased significantly with the progress of science, it is still particularly important to strengthen the prevention and control of fasciolosis. Among the anticipated factors, the type of season showed a statistically significant association with fasciolosis. Thus, more attention should be paid when sheep and goats are grazing in spring and summer, and researchers should monitor the quality of drinking water, as doing so is conducive to reducing economic losses.

## Figures and Tables

**Table 1:** Pooled prevalence of ovine and caprine fasciolosis in China.

	No. Studies	No. Examined	No. Positive	% (95% Cl)	Heterogeneity			Univariate Meta-Regression	
					χ2	*p*-Value	*I^2^* (%)	*p*-Value	Coefficient (95% CI)
Region									
Central China	2	6773	270	4.87 [1.98–8.95]	1.80	*p* = 0.18	44.3	0.0747	−0.3511 (−0.7372 to 0.0350)
South China	5	8356	3725	25.92 [8.22–49.21]	489.94	*p* < 0.01	99.2	0.6160	−0.0649 (−0.3183 to 0.1886)
Southwest China	18	23,126	6456	25.13 [18.53–32.37]	1824.45	*p* = 0	99.1	0.3397	−0.0761 (−0.2323 to 0.0801)
Northwest China	30	77,149	17,531	31.89 [21.76–42.96]	22,917.04	*p* = 0	99.9	-	-
Northeast China	2	181,925	13,927	18.06 [1.47–47.10]	52.03	*p* < 0.01	98.1	0.4137	−0.1593 (−0.5413 to 0.2227)
North China	2	478	21	4.38 [2.73–6.39]	0.36	*p* = 0.55	0.0%	0.0521	−0.3797 (−0.7629 to −0.0034)
Southeast China	2	391	43	10.97 [7.87–14.51]	1.15	*p* = 0.28	12.8%	0.1857	−0.2584 (−0.6412 to 0.1243)
Altitude									
Low altitude	1	160	38	23.75 [17.50–30.63]	0.00	*p* = 0	-	0.8727	−0.0511 (−0.6757 to 0.5736)
High Altitude	6	42,306	5699	28.23 [10.14–51.08]	9330.24	*p* = 0	99.9	-	-
Rainfall									
≥800	2	10,009	1791	19.85 [14.48–25.85]	3.40	*p* = 0.07	70.6	-	-
<800	7	236,786	24,337	15.34 [4.95–30.07]	16,069.24	*p* = 0	100.0	0.7001	−0.0689 (−0.4193 to 0.2815)
Temperature									
<10	5	41,342	5146	13.75 [2.04–33.43]	8478.58	*p* = 0	100.0	0.6521	−0.0913 (−0.4880 to 0.3055)
10–20	2	10,009	1791	19.85 [14.65–25.63]	3.40	*p* = 0.07	70.6	-	-
Season									
Spring	5	75,550	5544	36.28 [15.32–60.45]	1439.19	*p* < 0.01	99.7	-	-
Summer	4	3183	552	11.45 [1.00–30.95]	754.46	*p* < 0.01	99.6	0.06862	−0.3016 (−0.6269 to 0.0237)
Autumn	5	36,747	2550	15.13 [3.58–32.70]	624.70	*p* < 0.01	99.4	0.1153	−0.2472 (−0.5549 to 0.0605)
Winter	**2**	**74,021**	**6828**	**5.44 [0.77–13.98]**	**61.36**	***p* < 0.01**	**98.4**	**0.0453**	**−0.4123 (−0.8160 to −0.0087)**
Sampling years									
2002–2011	26	29,293	9153	35.75 [28.15–43.73]	2647.99	*p* = 0	99.1	-	-
2012–2022	**21**	**253,080**	**28,210**	**20.63 [10.42–33.22]**	**23,060.23**	***p* = 0**	**99.9**	**0.0342**	**−0.1701 (−0.3275 to −0.0127)**
Age									
0–2	5	1938	651	22.24 [4.61–48.04]	183.64	*p* < 0.01	97.8	0.5509	−0.1111 (−0.4764 to 0.2541)
>2	4	18,880	8076	32.26 [13.91–54.04]	142.44	*p* < 0.01	97.9	-	-
Sex									
Male	2	713	248	29.54 [15.89–45.38]	9.50	*p* < 0.01	89.5	0.1239	−0.1954 (−0.4442 to 0.0535)
Female	3	18,977	8180	48.33 [32.07–64.78]	33.10	*p* < 0.01	94.0	-	-
Host									
Sheep	22	68,473	14,823	34.74 [23.74–46.63]	20,121.31	*p* = 0	99.9	-	-
Goat	16	35,525	11,861	25.41 [15.09–37.36]	3208.48	*p* = 0	99.5	0.2675	−0.1019 (−0.2820 to 0.0782)
Feeding model									
Free range	20	228,303	19,010	26.83 [13.70–42.48]	8082.32	*p* = 0	99.8	-	-
Stall-feed	4	1140	302	11.83 [0.09–38.74]	422.59	*p* < 0.01	99.3	0.3318	−0.1934 (−0.5841 to 0.1972)
Half feed	2	491	119	25.32 [7.53–49.12]	30.77	*p* < 0.01	96.8	0.9510	−0.0166 (−0.5454 to 0.5122)
Species									
*Fasciola hepatica*	54	290,784	38,912	27.25 [20.51–34.57]	36,434.36	*p* = 0	99.9	-	-
*Fasciola gigantica*	**2**	**267**	**3**	**1.09 [0.20–2.68]**	**0.42**	***p* = 0.52**	**0.0%**	**0.0457**	**−0.4282 (−0.8482 to −0.0082)**
Diagnostic method									
Microscopy	35	234,078	22,660	30.16 [20.91–40.31]	15,837.03	*p* = 0	99.8	0.4075	−0.1430 (−0.4814 to 0.1954)
Immunological test	3	8792	4016	43.12 [35.72–50.68]	37.93	*p* < 0.01	94.7	-	-
Molecular	1	107	27	25.23 [17.50–33.86]	0.00	-	-	0.5518	−0.1978 (−0.8493 to 0.4537)
Slaughter	20	47,546	14,788	18.11 [11.03–26.49]	10,191.70	*p* = 0	99.8	0.1089	−0.2849 (−0.6332 to 0.0634)

## Data Availability

All datasets are included in the manuscript or as supplementary files.

## Supplementary Materials

The following supporting information can be downloaded at: https://www.mdpi.com/article/10.3390/ani13101687/s1, Figure S1: Sensitivity analysis (“Omitting” means if the article was omitted during the analysis process); Figure S2: Forest plot of the prevalence of ovine fasciolosis in the studies conducted in China; Table S1: Studies included in the analysis of ovine fasciolosis in China. References [43,44,45,46,47,48,49,50,51,52,53,54,55,56,57,58,59,60,61,62,63,64,65,66,67,68,69,70,71,72,73,74,75,76,77,78,79,80,81,82,83,84,85,86,87,88,89,90,91,92,93,94,95,96,97,98,99,100] are cited in Supplementary Materials.
