# Supplementary material for: Prevalence and Risk Factors of Ovine and Caprine Fasciolosis in the Last 20 Years in China: A Systematic Review and Meta-Analysis
Source: Animals (Basel). 2023 May 18;13(10):1687. doi: 10.3390/ani13101687 (PMC10215759; doi:10.3390/ani13101687)
Supplement: Supplementary file 1 [file animals-13-01687-s001.zip › Table S1.pdf]

Table S1. Studies included in the analysis of ovine and caprine fasciolosis in China.

Study	Host	Sampling years	Area	*Fasciola* species	Rainfall	Temperature	Altitude	Season	Stage	Sex	Feeding mode	Detect	Sample method	Number	Case	Score	Quality
Ai et al (2021) [43]	/	2017	Xingjiang	H	N	N	N	N	N	N	Y	Mic	R	138	128	4	High
Ai and Su (2014) [44]	/	2011	Xinjiang	H	Y	N	N	Y	N	N	N	S	R	2,300	983	4	High
Ai et al (2013) [45]	/	2013	Yunnan	H	N	N	N	N	N	N	N	MB	/	107	27	2	Middle
Bao (2014) [46]	Sheep	2014-2016	Xinjiang	H	N	N	N	N	N	N	N	-	R	1,200	18	3	Middle
Cai (2012) [47]	Sheep	2009	Qinghai	H	N	N	N	N	N	Y	N	Mic	/	225	105	3	Middle
Chen (2006) [48]	Sheep	2004-2005	Qinghai	H	N	N	N	N	N	N	Y	Mic	/	30	20	2	Middle
Cuo (2012) [49]	Sheep	2011	Qinghai	H	N	N	N	Y	N	N	N	Mic,S	R	142	29	4	High
Deng et al (2006) [50]	Sheep	2004	Henan	*Fasciola*	N	N	N	Y	N	N	N	S	/	46	4	3	Middle
Ding (2013) [51]	Sheep	2012	Qinghai	H	Y	Y	Y	Y	N	N	N	Mic	/	310	8	4	High
Gao et al (2020) [11]	Sheep	2012-2017	Guangxi, Tibet,Sichuan	H	N	N	N	N	N	Y	Y	I	/	1,092	405	4	High
Guo (2008) [52]	Sheep	/	Qinghai	H	N	N	N	N	N	N	N	Mic,S	/	50	19	2	Middle
Guo et al (2003) [53]	Goat	/	Shaanxi	H	N	N	N	N	N	N	N	/	/	70	54	1	Low
He (2014) [54]	Goat	2004	Yunnan	H	N	N	N	N	N	N	N	/	R	2,120	742	3	Middle
He et al (2006) [55]	/	2004-2005	Yunnan	/	N	N	N	N	N	N	N	Mic	/	437	198	3	Middle

Hu (2016) [56]	/	2015	Heilongjiang	H	Y	N	N	Y	N	Y	Y	Mic	/	181,801	13,887	4	High
Hu et al (2016) [57]	/	/	Qinghai	H	N	N	N	N	N	N	Y	Mic,S	/	1,252	1	2	Middle
Huang et al (2021) [58]	/	2018-2019	Hubei	H	N	N	N	N	N	N	N	S	/	6,727	266	3	Middle
Huang et al (2015) [59]	/	2012	Hebei	/	N	N	N	N	Y	N	M	Mic	/	368	15	3	Middle
Kan et al (2016) [60]	/	2014	Qinghai	H	N	N	N	N	N	N	Y	Mic	R	226	1	4	High
La et al (2014) [61]	/	2013	Tibet	H	N	N	N	Y	N	N	Y	S	/	35	7	3	Middle
Li (2014) [62]	/	2012-2013	Qinghai	H	N	N	N	N	N	N	N	Mic	/	1,110	431	3	Middle
Li (2013) [63]	Goat	2008-2011	Guizhou	H	Y	Y	N	N	N	N	N	/	/	9,849	1,753	3	Middle
Li and Lv (2011) [64]	Sheep	2010	Qinghai	/	N	N	N	Y	N	N	Y	Mic	R	529	260	4	High
Li and Ma (2009) [65]	Sheep	2009	Qinghai	H	N	N	N	Y	Y	N	Y	Mic	/	300	168	4	High
Li et al (2007) [66]	/	2006	Yunnan	H	N	N	N	N	N	N	N	Mic,S	/	517	258	3	Middle
Lin et al (2016) [67]	Goat	2014-2015	Fujian	*Fasciola*	N	N	N	N	N	N	N	S	/	221	21	3	Middle
Liu (2014) [68]	Goat	/	Hunan	H	N	N	N	N	N	N	N	S	R	68	23	3	Middle
Liu et al (2021) [69]	Sheep	2016-2020	Gansu	H	Y	N	N	Y	Y	Y	N	S	/	18,373	7,918	4	High

Liu et al (2004) [70]	Goat	2003	Guizhou, Chongqing	H	N	N	N	Y	N	N	N	S	R	5,957	2424	5	High
Ma (2015) [71]	Sheep	2013	Qinghai	H	N	N	N	N	N	N	Y	Mic	/	100	85	3	Middle
Ning (2016) [72]	Sheep	/	Xinjiang	H	N	N	N	Y	N	N	Y	S	/	4,070	142	3	Middle
Nu and Sai (2011) [73]	/	/	Xinjiang	H	N	N	N	N	N	N	Y	S	/	236	5	2	Middle
Pan (2019) [74]	Goat	/	Guangxi	H	N	N	N	N	N	N	N	Mic	/	315	14	2	Middle
Ren (2016) [75]	Sheep	2015	Qinghai	H	N	N	N	N	N	N	Y	Mic	R	531	264	4	High
Ren (2016) [76]	Sheep	2016	Qinghai	H	Y	Y	Y	Y	N	N	Y	Mic	/	32,726	1,271	4	High
Shang and Wang (2010) [77]	Sheep	2008	Qinghai	H	N	N	N	Y	N	N	N	Mic	R	428	207	4	High
Shi (2009) [78]	Sheep	2008	Qinghai	H	Y	Y	Y	N	N	N	N	S	R	1,242	268	5	High
Tao (2017) [79]	Sheep, Goat	/	Qinghai	H	N	N	Y	N	N	N	N	Mic	/	7,030	3,597	3	Middle
Tao et al (2010) [80]	Goat	2006-2007	Guangxi	H	N	N	N	N	N	N	N	S	/	152	11	3	Middle
Wang (2009) [81]	Sheep	2008	Qinghai	H	N	N	Y	Y	N	N	N	Mic	/	141	89	4	High
Wang (2007) [82]	Sheep, Goat	/	/	H	Y	Y	Y	N	N	N	Y	S	/	34	2	3	Middle
Wang (2007) [83]	Goat	2006	Yunnan	H	N	N	N	Y	N	N	N	Mic	/	948	99	3	Middle
Wang et al (2020) [84]	/	2019	Guizhou	H	N	N	N	N	N	N	N	Mic	/	286	78	3	Middle
Wang et al (2018) [85]	/	/	Hebei	H	N	N	N	N	N	N	N	Mic	/	110	6	2	Middle
Wang et al (2015) [86]	/	2014-2015	Jiangsu	H	N	N	N	N	N	N	Y	Mic	/	170	22	3	Middle

Wang et al (2005) [87]	Goat, Sheep	2003-2004	Heilongjiang	H	N	N	N	N	N	N	N	S	R	124	40	4	High
Wei et al (2021) [88]	Goat	2018-2019	Guangxi	/	N	N	N	N	N	N	N	I	/	7.650	3,587	3	Middle
Wei et al (2013) [89]	Goat	2011	Guizhou	H	N	N	N	Y	N	N	N	S	R	120	12	3	Middle
Wu (2012) [90]	/	2011	Qinghai	H	N	N	Y	Y	N	N	N	Mic	/	1,133	472	3	Middle
Xu and Wang (2011) [12]	Goat	2010	Qinghai	H	N	N	N	N	N	Y	Y	Mic	R	270	179	5	High
Yang et al (2014) [91]	/	2012-2013	Guizhou	/	Y	Y	Y	N	N	N	N	Mic	/	160	38	3	Middle
Yang and He (2011) [92]	/	2009	Yunnan	H	N	N	N	Y	N	N	N	Mic	/	874	396	3	Middle
Ye et al (2020) [93]	/	2008	Sichuan	H	N	N	N	Y	N	N	Y	Mic	/	289	44	3	Middle
Zeng and Cao (2014) [94]	/	/	Sichuan	H	N	N	N	N	N	N	Y	S	/	450	63	2	Middle
Zhai (2020) [95]	Goat	2018	Gansu	H	N	N	N	Y	Y	N	Y	Mic	R	422	43	5	High
Zhang (2019) [96]	Cattle	2006-2015	Qinghai	H	N	N	N	N	N	N	N	/	/	1,601	594	2	Middle
Zhang et al (2014) [97]	Goat	2013-2014	Sichuan	H	N	N	N	N	N	N	N	Mic	/	56	2	3	Middle
Zhao et al (2016) [98]	Sheep	/	Qinghai	H	N	N	N	N	Y	N	Y	Mic	/	604	193	3	Middle
Zhao et al (2011) [99]	Sheep	2010-2011	Qinghai	H	N	N	N	N	N	N	N	Mic	/	50	33	3	Middle

Zhou et al (2005) [100]	/	2002	Qinghai	H	N	N	N	Y	N	N	N	S	R	448	74	4	High

Footnote: Reference numbers in the Appendix were preceded by "A"; Y, Yes which refers to that there are relevant data in the subgroup; H refers to *Fasciola hepatica* and G refers to *Fasciola gigantica*; *Fasciola* refers to the data including *F. hepatica* and *F. gigantica*; N, no data; Mic, microscopy; S, slaughter; I, immunological test; MB, molecular biological assay; R, random; NR, not random

43. Ai, T.; Bi, S. Several trematodes of Kazakh sheep in the pastoral area of Ili valley investigation and prevention of infection. *Livestock Poult. Industry* **2021**, 32(6), 5-6 (In Chinese).
44. Ai, T.; Su, W. Investigation on *Fasciola hepatica* infection in Kazakh sheep. *Chin. J. Vet. Med.* **2014**, 4, 40 (In Chinese).
45. Ai, L.; Chen, M.X.; Lv, X.; Zang, W.; Zhu, T.J.; Xu, X.N.; Cai, Y.C.; Chen, SH.; Luo, J.J.; Chen, B.J.; Zhang, J.G.; Zhou, X.N.; Chen, J.X. Surveillance and molecular identification of *Fasciola* spp. from cattle and goats at Binchuan， Yunnan province. *J. Trop. Med.* **2013**, 13 (6), 791-794 (In Chinese).
46. Bao, W. Investigation on infection of *Fasciola hepatica* in this area. *J. Biotech World* **2014**, 12, 27 (In Chinese).
47. Cai, D.J. Epidemiological investigation of *Fasciola hepatica* in Tibetan sheep in high altitude pastoral areas. *Anim. Husb. Vet. Med.* **2012**, 44, 8 (In Chinese).
48. Chen, C.Y. Epidemiological investigation of *Fasciola hepatica* in sheep in Xianghua, Datong. *Qinghai J. Anim. Husb. Vet. Med.* **2006**, 36(4), 26 (In Chinese).
49. Cuo, M.J. Investigation on *Fasciola hepatica* of sheep in Maqin, Qinghai. *Chin. J. Vet. Med.* **2012**, 48(6), 77 (In Chinese).
50. Deng, W.; Pang, Y.Z.; Zhao, R.Q.; Geng, E.Q.; Zhang, H.J.; Zhao, S.J.; Zhang, S.X.; Wang, Z.H. Investigation on the infection of main parasites in digestive tract of henan big tail han sheep. *Acta Ecologiae Anim. Domastici* **2006**, 27(4),101-104 (In Chinese).
51. Ding, D.S. Investigation on the parasites in sheep in Xunhua. *New Countryside* **2013**, 14, 190-191 (In Chinese).
52. Guo, Z.H. Detection of *Fasciola hepatica* in Tibetan sheep by IHA. *Chin. J. Anim. Health inspect.* **2008**, 25(8), 36-37 (In Chinese).
53. Guo, X.Y.; Shen, W.Z.; Xue, Z.D. Research on parasitic fauna in guanzhong dairy goat in Guanzhong, Shaanxi. J. *Yangling Vocational Tech. College* **2003**, 2(2), 4-6 (In Chinese).
54. He, Z.C. Investigation and control measures of *Fasciola hepatica* in goats. *Biotech. World* **2014**, 11, 64 (In Chinese).
55. He, P.; Chen, Z.S.; Liu, G.B.; Shao, W.S.; Zhou, H.S.; Wang, X.M.; Feng, H.X. Epidemic law and diagnosis and treatment of sheep *Fasciola hepatica* in Dehong. *Yunnan Anim. Husb. Vet*. **2006**, 6, 30-31 (In Chinese).
56. Hu, Y.H. Investigation on the distribution of *Fasciola hepatica* infection in sheep in Keshan. *Primary Agri. Tech. Extent.* **2016**, 5, 106-108 (In Chinese).
57. Hu, G.W.; Zhao, Q.B.; Ma, Z.Q.; Kan, W.; Luo, J.J.; Cai, J.S. Epidemiological investigation of *Fasciola hepatica* in sheep in Dulan county. *Henan Anim. Husb. Vet.* **2016**, 37, 1 (In Chinese).
58. Huang, L.; Zhou, P.P.; Li, K.M.; Du, Z.Q. Status and quarantine measures of *Fasciola hepatica* in cattle and sheep in slaughterhouse. *Prev. Epid. Quarantine* **2021**, 1,142-143 (In Chinese).
59. Huang, Z.X.; Mi, T.G.; Zhao, X.; Zhang, B.Y. Investigation on helminth infection in sheep digestive tract in Handan. *Heilongjiang Anim. Husb. Vet.* **2015**, 3,67-69 (In Chinese).
60. Kan, W.; Zhao, Q.B.; Shen, Y.L.; Ma, Z.Q.; Hu, G.W.; Li, J.; Sun, S.J.; Ma, R.L.; Cai, J.S. Investigation on *Fasciola hepatica* infection in sheep in Dulan, Qinghai. *Henan Anim. Husb. Vet.* **2016**, 37(4), 12-13 (In Chinese).
61. La, B.C.D.; Wu, J.C.M.; Lin, H.L.; Ma, X.B. Investigation and integrated control techniques of *Fasciola hepatica* in cattle and sheep in a county of Xigaze, Tibet. *Tibet Sci. Tech.* **2014**, 1, 50-52 (In Chinese).
62. Li, H.X. Investigation and analysis on the distribution of *Fasciola hepatica* in Geermu. Chin. *Qinghai J. Anim. Vet. Sci.* **2014**, 44(3), 25 (In Chinese).
63. Li, M.Z. Epidemiological investigation and control of goat parasitic diseases in Jiangkou. *Guizhou Anim. Husb. Vet*. **2013**, 37(4), 26-27 (In Chinese).
64. Li, H.Q.; Lv, W.H. Investigation on *Fasciola hepatica* infection in sheep in Gonghe. *Shandong Anim. Husb. Vet.* **2011**, 32, 3 (In Chinese).

65. Li, S.S.; Ma, Y.L. Investigation of *Fasciola hepatica* in sheep. *Chin. Livestock Breed.* **2009**, 5 (In Chinese).
66. Li, W.X.; Qian, L.D.; Hu, X.J.; Li, X.Z. Epidemic situation and integrated control measures of sheep *Fasciola hepatica*. *Contemp. Anim. Husb.* **2007**, 11, 20-21 (In Chinese).
67. Lin, L.; Jiang, B.; Wu, S.H.; Zhang, S.Z.; Lin, S.; Cai, X. Helminthic infection on goats in Fujian. *Fujian J. Agri. Sci.* **2016**, 31(6), 575-579 (In Chinese).
68. Liu, B.C. Etiological investigation on goat parasitic diseases in Xiangxi. *Hunan J. Anim. Sci. Vet. Med.* **2014**, 5, 26-28 (In Chinese).
69. Liu, Y.D.; Zhang, R.X.; Li, B.J. Investigation on infection with sheep *Fasciola hepatica* in Lintao county and its surrounding regions in Gansu province. *Chin. Anim. Quarantine* **2021**, 38, 9 (In Chinese).
70. Liu, Y.; Zhang, J.; Chen, W. Investigation on infection of *Fasciola hepatica* and *Plasmodium Pancreaticum* in slaughtered sheep in Zunyi. *Chin. Anim. Health Inspec*t.**2004**, 21(2), 33 (In Chinese).
71. Ma, D.L. Investigation on *Fasciola hepatica* of Tibetan sheep in Datan. *Guizhou J. Anim. Husb. Vet. Med.* **2015**, 39(3), 36 (In Chinese).
72. Ning, Z.S. Investigation on infection of *Fasciola hepatica* in Balikun. *Xinjiang Anim. Husb.* **2016**, 3, 35-36 (In Chinese).
73. Nu, L.M.; Sai, B.T. Investigation and analysis on epidemic situation of sheep *Fasciola hepatica*. *Xinjiang Anim. Husb.* **2011**, S1, 29 (In Chinese).
74. Pan, H.X. Parasitic infection and control strategy of goat in Wuxuan. *Livestock Poultry Indus.* **2019**, 30(8), 101-102 (In Chinese).
75. Ren, Q.Z.M. Investigation on infection of sheep *Fasciola hepatica* in Qinghai. *Shandong Anim. Husb. Vet.* **2016**, 37(10), 49 (In Chinese).
76. Ren, Z. Investigation on *Fasciola hepatica* infection in sheep in Gonghe. *Contemp. Anim. Husb*. **2016**, 87 (In Chinese).
77. Shang, Q.S.; Wang, H.G. Epidemiological investigation of sheep *Fasciola hepatica* in Hualong, Qinghai. *Chin. J. Vet. Med*. **2010**, 46(7), 33 (In Chinese).
78. Shi, W.Y. Investigation on *Fasciola hepatica* infection in sheep in Wulan. *Contemp. Anim. Husb.* **2009**, 10,17-18 (In Chinese).
79. Tao, L.D. Epidemic situation and control effect of sheep *Fasciola hepatica* in Shinaihai, Gonghe. *Shandong J. Anim. Sci. Vet. Med.* **2017**, 38(7), 73 (In Chinese).
80. Tao, L.; Wei, Z.F.; Lan, M.Y.; Li, J.; Nong, Q.W.; Huang, M.X.; Wei, Q.Z.; Ji, A.H.; Wei, H.Q.; Yang, W.; Chen, Z.X. Epidemiological investigation of main goat diseases in Guangxi. *Chin. Anim. Husb. Vet. Med.* **2010**, 37(2), 138-140 (In Chinese).
81. Wang, X.H. Investigation on the parasites in Tibetan sheep in Guinan. *Heilongjiang Anim. Sci. Vet. Med.* **2009**, 20, 80 (In Chinese).
82. Wang, Q. Report about infection of sheep parasite in Tianzhu Zang automomous county. *Chin. J. Vet. Parasit.* **2007**, 15(3), 39-41 (In Chinese).
83. Wang, Y.Q. Investigation and treatment of *Fasciola hepatica* in cattle and sheep in Yangbi, Yunnan. *Chin. J. Vet. Parasit.* **2007**, 15 (2), 27-29 (In Chinese).
84. Wang, J.P.; Yu, S.J.; Wang, J.P.; Yu, X.W.; Pan, W. Infection of *Taenia solium*, *Pulmonary Filariae*, *Fasciola hepatica* and *Haemorhabditis contortus* in Leishan in 2019. *Guizhou Anim. Husb. Vet.* **2020**, 44(2), 42-46 (In Chinese).
85. Wang, R.S.; Li, Z.; Xu, Y.G.; Hao, L.B.; Fu, C.F.; Xue, Z.G.; Zhao, T. Investigation and control of *Fasciola hepatica* in mutton sheep in agricultural areas. *Today Anim. Husb. Vet.* **2018**, 34(5), 75 (In Chinese).
86. Wang, J.; Pan, W.; Jin, L.M.; Chen, Z.L.; Xu, X.J.; Xue, C.H. The Investigation of infection in sheep gastrointestinal parasites. *J. Jinling Institute Tech.* **2015**, 31(4), 89-92 (In Chinese).
87. Wang, C.R.; Ma, G.F.; Zhao, J.P.; Wang, Z.F.; Liu, X.L.; Liu, W.; Gong, X.J. Investigation and control technique on parasites of sheep in the western of Heilongjiang province. *J. Heilongjiang Bayi Agri. Univ.* **2005**, 17(4),53-57 (In Chinese).
88. Wei, X.M.; Liao, A.C.; Chen, X.; Li, J.C.; Huang, Y.S.; He, F.Y.; Wei, J.H.; Xie, T. Seroprevalence investigation on goat *Fasciola hepatica* in Hechi of Guangxi from 2018 to 2019. *Chin. Anim. Quarantine.* **2021**, 38(7), 21-24 (In Chinese).
89. Wei, C.K.; Liu, B.; Fan, Z.X. Investigation on goat parasitic diseases in Weng'an. *Hubei J. Anim. Vet. Sci.* **2013**, 34(7), 49-50 (In Chinese).
90. Wu, P. Investigation on the control of *Fasciola hepatica* in Mongolian sheep in Wutumeiren. *Chin. Qinghai J. Anim. Vet. Sci.* **2012**, 42(4), 35 (In Chinese).
91. Yang, S.Q.; Shi, C.Q.; Jian, W.X.; Yang, G.Y.; Huang, S.X. Investigation on cattle and sheep parasites in Yuping. *Guizhou Anim. Husb. Vet.* **2014**, 38(6), 29-32 (In Chinese).

92. Yang, R.F.; He, P. Epidemic law, diagnosis and control of *Fasciola hepatica* in Longling. *Anim. Breed. Feed* **2011**, 3, 22-23 (In Chinese).
93. Ye, Y.G.; Xiao, L.; Wei, Y.; Kang, R.M.; Yu, J.F.; Zhang, T.; Ye, J.Q.; Cao, Y.; Xie, J.; Li, X.Y.; Pan, M.; Lin, Y.; Sun, W.P.; Dai, Z.J.; Liao, D.J. The survey of gastrointestinal helminths infection in half shed-feeding sheeps and goats in some areas of Sichuan province. *Chin. J. Vet. Med.* **2020**, 56(8), 1-6 (In Chinese).
94. Zeng, R.Q.; Cao, H.Z. Epidemiology of fascioliasis of sheep liver in Baimo River area of Qionglai city. *Heilongjiang Anim. Sci. Vet. Med.* **2014**, 10,74-76 (In Chinese).
95. Zhai, J.Y. Epidemic survey of *Fasciola hepatica* in goats in Suzhou, Jiuquan. Chin. *Herbivore. Sci.* **2020**, 40(2), 85-86 (In Chinese).
96. Zhang, P. Epidemiological investigation of *Fasciola hepatica* in cattle and sheep in Qinghai. *Vet. Guide* **2019**, (15), 27-28 (In Chinese).
97. Zhang, W.L.; Hao, G.Y.; Luo, Q.H. Infection status of gastrointestinal parasite in Huili black goat. *Anim. Husb. Vet. Fishery Silkworm*. **2014**, 42(7),107-110 (In Chinese).
98. Zhao, C.Q.; Guo, M.J.; Li, W.; Chen, G.; Kang, M. Investigation on prevalence of *Fasciola hepatica* infection in sheep in some areas of Qinghai. *Anim. Husb. Vet. Med.* **2016**, 48(3), 134-136 (In Chinese).
99. Zhao, Y.L.; Zeng, Z.H. Epidemiological investigation and control of *Fasciola hepatica* in sheep. *Shandong J. Anim. Sci. Vet. Med*. **2011**, 32(11), 53-54 (In Chinese).
100. Zhou, J.; Xiao, F.; Xiao, H. Investigation on sheep fascioliasis in Dulan. Qinghai J. *Anim. Sci. Vet. Med*. **2005**, 35(4), 30 (In Chinese).
