# Supplementary figures and images for: Prevalence and Risk Factors of Ovine and Caprine Fasciolosis in the Last 20 Years in China: A Systematic Review and Meta-Analysis
Source: Animals (Basel). 2023 May 18;13(10):1687. doi: 10.3390/ani13101687 (PMC10215759; doi:10.3390/ani13101687)

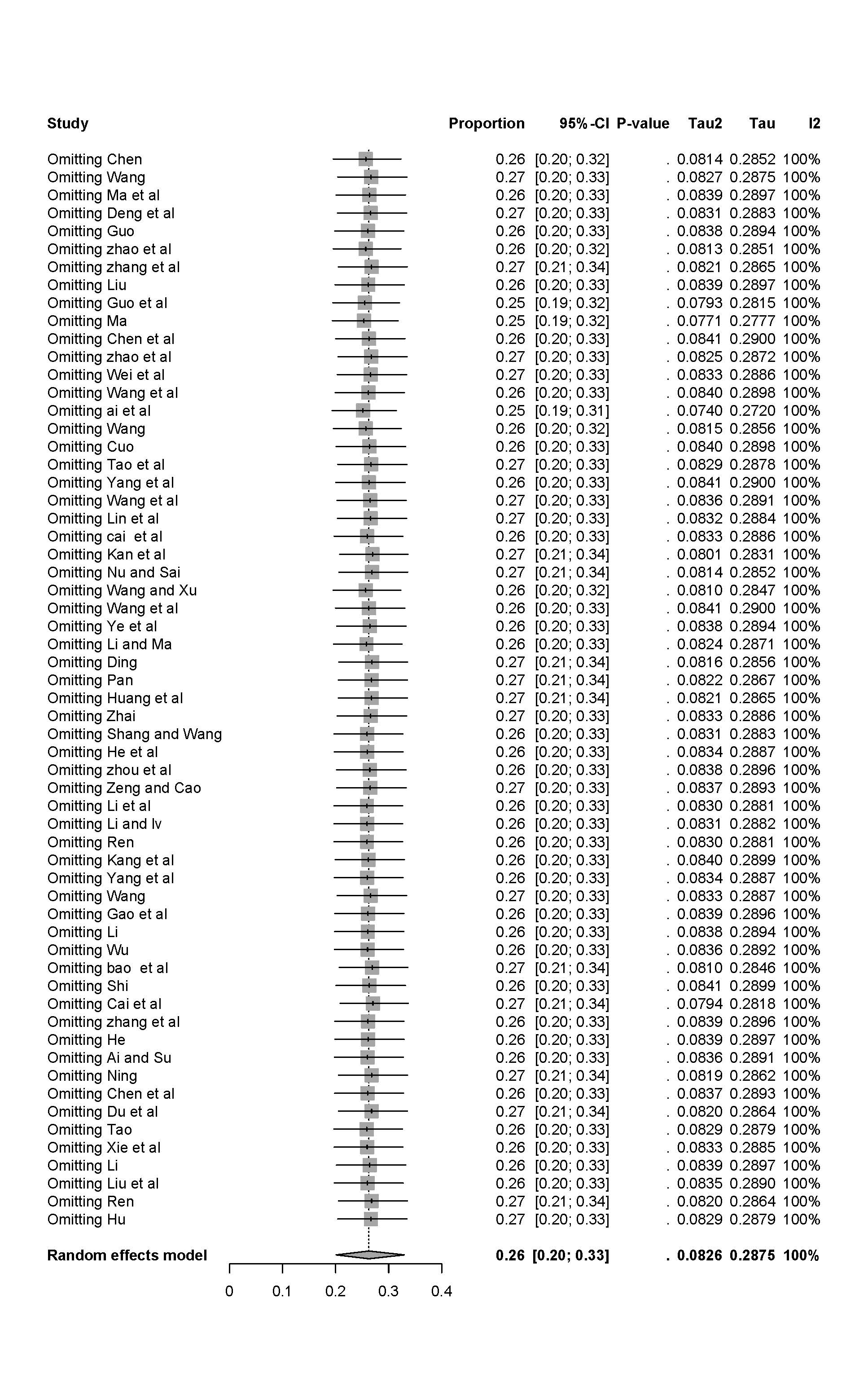

Supplement: Supplementary file 1 [file animals-13-01687-s001.zip › Figure S1.jpg]

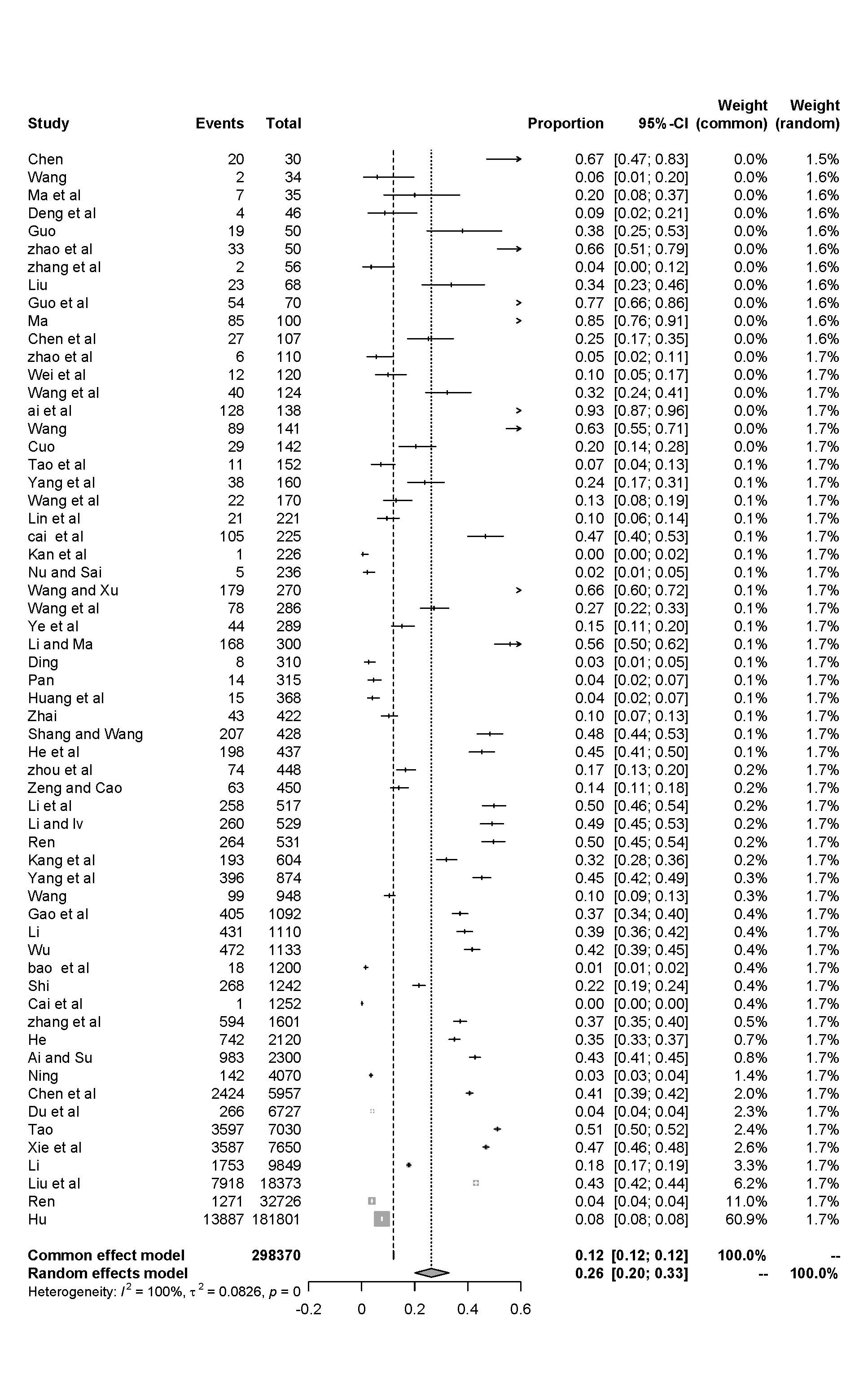

Supplement: Supplementary file 1 [file animals-13-01687-s001.zip › Figure S2.jpg]
